# Studies on the Evolution of Fatigue Strength of Aluminium Wires for Overhead Line Conductors

**DOI:** 10.3390/ma17112537

**Published:** 2024-05-24

**Authors:** Bartosz Jurkiewicz, Beata Smyrak

**Affiliations:** Faculty of Nonferous Metals, AGH University of Krakow, 30-059 Krakow, Poland

**Keywords:** overhead conductors, ACSR, aluminium conductor steel reinforced, fatigue strength, fatigue failure

## Abstract

Traditional ACSR overhead wires, which consist of a high-strength steel core and several layers of aluminium wires, are currently the most popular overhead line conductor (OHL) design globally. Operating conditions, particularly operating under varying stresses from Karman vortices, lead to the fatigue cracking of wires of the outer layer, followed by wires of the inner layers. Karman vortices are formed by the detachment of a laminar wind stream flowing around the conductor, which causes vibrations in the conductor called wind or aeolian oscillations. Aluminium wires are manufactured using standard batch material drawing technology. Although the fatigue strength of such wires is not standardised, there are various criteria for evaluating this characteristic, as well as established limits on the number of cycles needed to break the first wires of the outer layer. Fatigue strength also strongly depends on the geometric structure of the wire and its operating conditions. The article analyses the influence of the mechanical condition of aluminium wires used in ACSR cables on their fatigue strength. We then present results from aluminium wire fatigue tests conducted on a specially constructed test rig. In addition, fatigue cracks were interpreted using scanning microscopy.

## 1. Introduction

Fatigue damage to overhead conductors (OHL) is one of the most important factors limiting the lifespan of transmission lines. Fatigue damage to conductors is usually caused by a combination of static loads (tensile load, clamping force, and span weight) and static loads.

Overhead power lines are constantly subjected to varying wind loads, which can gradually weaken their durability and, consequently, shorten their service life. Wind forces cause three main types of conductor vibrations:

- Aeolian vibrations, characterised by frequencies from 3 to 150 Hz and amplitudes smaller than the diameter of the conductor;
- Galloping, which occurs with frequencies from 0.1 to 1 Hz and amplitudes ranging from ±0.1 to 1 times the sag of the conductor;
- Tracking-induced vibrations, which occur in a frequency range from 0.15 to 10 Hz, with an amplitude of 0.5 to 80 times the diameter of the conductor.

Among the different types of vibrations, aeolian vibrations, which are typical of lowland areas, are particularly relevant under Polish conditions. Depending on the location of the transmission line, the conductor of a power line during operations is exposed to various atmospheric conditions, including the varying effects of wind, which induce oscillatory movements around the conductor, ultimately leading to cyclic vibrations and, consequently, causing fatigue damage to the conductors in various layers. Cable damage most often occurs at points of restricted movement, such as areas in contact with passing or suspended terminals. The conductors in the outer layer are the first to crack, followed by the conductors in subsequent layers [1,2,3,4].

Aeolian vibrations are characterised by a low amplitude (outside the range where resonance occurs) and a very high frequency. High-voltage lines are most susceptible to aeolian vibrations because of the large distances between their supporting poles. Fatigue failure of a wire is the result of classic fatigue and is further accelerated by corrosion, which involves wear between the moving surfaces of the wire. Fatigue failure can occur in any material if the combination of static and dynamic stresses exceeds the fatigue strength of the loaded material [5,6]. For a wire, instantaneous loads include normal axial and radial stresses, as well as bending, shear, and torsional stress. The number of cycles in alternating loads that can be transferred without damaging the conductor depends on the value of the static load, the amplitude of dynamic stresses, and the intensity of frictional corrosion. In particular, the mechanism of fatigue damage in power line conductors is based on the induction of relative movements at the (a) wire-to-wire, (b) wire-to-clamp, and (c) wire-to-support contact planes, which are caused by the cyclic movement of the conductor. At the same time, frictional corrosion is catalysed by the action of stresses and bending moments, leading to cracks and surface wire propagation and, ultimately, yielding mechanical failure of the wire [7,8,9].

In a multilayer structure composed of wires, the breakage of one wire (usually from the outer layer) due to fatigue will rapidly cause the entire conductor to rupture [10].

Importantly, multilayer constructions are favourable from the perspective of fatigue. In such constructions, the fracture of individual wires leads to only a slight increase in the stress of the wire. The intersection points of the horizontal dashed line representing the highest permissible working stresses of the wire with the stress increase curves above EDS (everyday stress) for the different designs correspond to static wire rupture. However, fretting processes are intensified in multilayer structures [11,12,13].

Therefore, the problem of fatigue failure must be considered when designing conduits. Fatigue life has traditionally been evaluated using cumulative damage methods or strength criteria related to the cyclic bending amplitude of the conductors. These methodologies use stress-cycle-to-failure curves dependent on conductor clamp assemblies obtained in resonance stand fatigue tests, according to specific standards, which require long test times, as well as special and expensive laboratory equipment. The Conseil International des Grands Réseaux Électriques (CIGRÉ) introduced the concept of everyday stress (EDS) into the design procedure for overhead conductors. EDS is defined as the maximum stress in a conductor under fixed-temperature conditions for a period of one year, without risk of damage from aeolian vibrations. The EDS value is expressed as the stress in the conductor during its longest service life (that is, the average stress) and is related to the conductor stress at 10–16 °C based on the 10-year creep of the conductor. EDS is often expressed as a percentage of the cable’s tensile strength. The EDS parameter was first proposed by CIGRÉ in 1960 [14,15,16]. Despite the introduction of this criterion, numerous failures of overhead conductors due to aeolian vibrations continue to be observed. For example, a CIGRÉ study showed that 78% of the lines failed due to fatigue damage within 20 years after being put in service [15].

**Figure 1:**
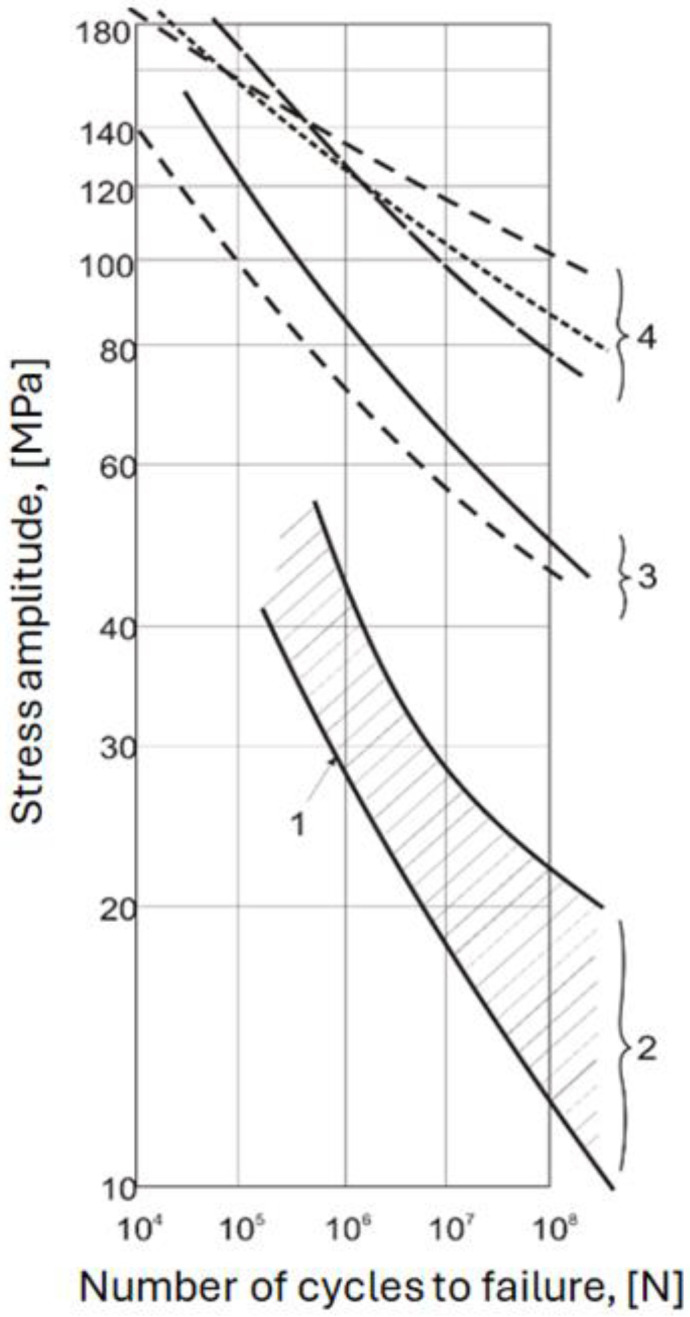
S–N curves for wires and cables on a logarithmic scale [17].

Due to the difficulties and high costs associated with the fatigue testing of conductors, the method of safe limit line, known as the CIGRÉ Safe Limit Line (CSBL), was proposed. CSBL is an S–N curve (number of stress cycles) constructed from various fatigue tests conducted on different conductor structures such as aluminium conductors and wires [16]. Figure 1 shows σ–N curves or the relationship between the amplitude of the alternating stress and the number of cycles, which is a widely accepted method for evaluating the fatigue resistance of materials [14].

Curves (4) and (3) represent wires (4-AlMgSi alloy, 3-Aluminium), the hatched area (2) represents conductors, and the lower line (1) represents the established CIGRÉ safe limit line (SBL). Compared to conductors, wires can withstand much higher stresses before fatigue failure (for the same number of vibration cycles). The lower position of the σ–N curves for the conductors is due to cyclic sliding, continuous refreshing, and oxidation of the contact surfaces between vibrating wires within and between layers. Thus, for the same amplitude of alternating stresses, conductors undergo fatigue failure at a much lower number of cycles than the individual wires from which the conductor is made [18,19].

Currently, there are two dominant methods used globally to reduce the risk of fatigue damage to overhead conductors caused by aeolian vibrations: the active method and the passive method. The passive method involves reducing stresses in the conductors, which does not help ensure high line capacity. Active protection, on the other hand, involves the use of vibration dampers installed on the overhead conductor. This solution, introduced to overhead lines in 1924, dominates most applications in overhead power systems. Another method for minimising the effects of aeolian vibrations is to use self-damping conductors such as ACSR-VR or oval spiral conductors such as ACSR-SE. However, due to the high complexity of manufacturing and installation technology, these conductors are not widely used [7,20,21,22,23].

The basic elements of OHL cables are wires, most often aluminium, whose properties determine the properties of the OHL cables, including their fatigue strength.

The fatigue strength of materials depends on a variety of factors, with material properties such as static strength, surface quality, structural state (grain size), and residual stresses playing key roles. Additionally, fatigue strength is affected by the conditions of the fatigue process, including the magnitude and direction of applied stress, stress amplitude, temperature, and duration of the process. Therefore, evaluating fatigue strength requires the consideration of many factors. Furthermore, the analysis of high-cycle fatigue processes must consider the evolution of changes in the mechanical properties of materials that can occur during long-term exposure to varying cyclic loads. The authors in [24] showed that for annealed aluminium (temper O), the material is strengthened in the early stages of fatigue when the volume of dislocation density in the material increases, accompanied by a forced slip band. However, in the case of strain-hardened materials such as cold-rolled aluminium (tempera H14), hardness is lower in the early stages of fatigue due to a reduction in the density of the dislocation [23,24,25].

In another study [26], researchers investigated the effect of surface quality on the fatigue strength of steel components. This study consisted of shooting the surfaces of the components, determining the roughness of the parameters, and establishing a relationship between the roughness parameter Ra and the number of damage cycles. This study clearly showed a correlation between surface quality and the number of cycles to failure. Many studies on the effects of surface quality on fatigue strength have focused on the development of surface modification methods to reduce the risk of fatigue crack initiation. The authors in [26] conducted fatigue tests of copper-coated aluminium specimens and demonstrated the beneficial ability of Cu coating to increase the number of cycles to fatigue failure.

Residual stresses also have a significant effect on fatigue strength, as outlined in [27], which found that compressive residual stresses have a significant positive effect on improving the fatigue strength of materials, while tensile residual stresses adversely affect strength properties.

In general, most previous research has focused on analysing the fatigue strength of steel products. Fewer studies, however, have addressed the issue of fatigue in nonferrous metals, particularly aluminium and its alloys. Given the usage trends of aluminium and its alloys in various applications, such as energy systems and in the automotive, aerospace, construction, and packaging industries, fatigue strength analysis is increasingly important. Given that structural aluminium alloys are mostly fatigue-resistant, fatigue strength and mechanical condition are also critical factors [28].

Aluminium wires are made using standard batch material drawing process technology. For overhead wires, the basic feedstock is an aluminium wire rod from a continuous Properzi line with a diameter of 9.5 mm in various mechanical states. This type of wire must have a minimum aluminium content of 99.5 or 99.7% by weight, depending on the grade. Wires must also offer adequate electrical conductivity and tensile strength. The rheological resistance and heat resistance of aluminium wires intended for the construction of high-voltage power cables are also limited by guidelines. Conversely, the fatigue strength of wires is not standardised. Nevertheless, there are various criteria for assessing this characteristic, as well as established limits on the number of cycles until the first wires in the outer layer break. Fatigue strength also depends enormously on the geometric structure of the wire and the conditions of its operation. The basic component of the conductor is its wires, most often aluminium wires, whose properties determine the properties of the entire conductor, including its fatigue strength. In general, fatigue strength can be changed via metallurgical intervention, i.e., by blending an aluminium alloy that guarantees an increase in strength and thus an increase in fatigue strength. Fatigue strength is also determined by the mechanical condition of the material, the quality of its surface, and the stress level of the wires.

Therefore, the objective of this study was to determine the effect of the mechanical condition of aluminium wires on their fatigue strength.

## 2. Materials and Methods

This study focused on fatigue tests of aluminium wires with different levels of strain hardening (12, 35, 70, and 90%). Based on the results, we determined the effect of strain hardening on the fatigue strength of aluminium wires commonly used for ACSR cables.

### 2.1. Fatigue Testing Materials

The aluminium wires subjected to fatigue strength tests in various mechanical states were manufactured under industrial conditions, with a wire drawing speed of 12 mm/s, specific die geometry (die angle = 18° and lubricants for the aluminium wire drawing process. Special dies were used in the production process, which guaranteed the best surface quality and minimum residual stresses of the wires tested.

Table 1 shows the chemical composition of the aluminium wires tested, and Table 2 shows the mechanical and electrical properties of the wire rod and the wires produced from it.

Due to the fact that the wires for fatigue testing should have the same diameter (approx. 3.0 mm), as dictated by the construction of the fatigue stand, a procedure was developed for preparing wires with a diameter of 3 mm at various levels of strain hardening. The procedure involved the process of wiredrawing wires with different initial diameters to wires with a final diameter of 3 mm. Wires of different initial diameters were pre-annealed to achieve the same level of mechanical properties. The level of strain hardening of wires after wiredrawing process is presented as engineering strain ($\varepsilon_{A}$) and true strain ($\varepsilon_{t}$) according to the following formulas:

Engineering strain ($\varepsilon_{A}$) is calculated by:

$$\varepsilon_{A}=\frac{A_{0}-A_{1}}{A_{0}}=\frac{{d_{0}}^{2}-{d_{1}}^{2}}{{d_{0}}^{2}}$$

where

$A_{0}$—cross section of wire before wiredrawing process.
$A_{1}$—cross section of wire after wiredrawing process.
$d_{0}$—diameter of wire before wiredrawing process.
$d_{1}$—diameter of wire after wiredrawing process.

True strain ($\varepsilon_{t}$) is calculated by:

$$\varepsilon_{t}=\ln\left(\frac{A_{0}}{A_{1}}\right)=\ln{\left(\frac{d_{0}}{d_{1}}\right)}^{2}$$

Relationship between $\varepsilon_{t}$ and $\varepsilon_{A}$ is calculated by:

$$\varepsilon_{t}=\ln\left(\frac{1}{1-\varepsilon_{A}}\right)$$

### 2.2. Fatigue Test Procedure

Fatigue strength tests were carried out on a dedicated testing apparatus. To prepare specimens for fatigue testing, we created straight specimens about 400 mm in length, which were then mounted on a rotary bending fatigue testing apparatus. The specimens were subjected to stresses of 129, 71, and 43 [MPa].

**Figure 2:**
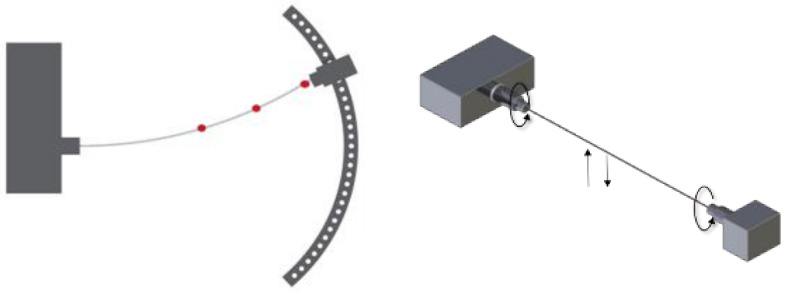
Diagram of the fatigue test stand.

The rotary bending fatigue testing device was designed to induce varying stresses in the material by bending the specimen. The specimen (wire) was subjected to axial rotary motion at 3000 revolutions per minute (RPM). As shown in Figure 2, stress modification was achieved by symmetrically bending the specimen to a known deflection:

$$\sigma_{g}=\frac{3\cdot E\cdot d\cdot y}{2\cdot L^{2}}$$

where $\sigma_{g}$ represents the bending stresses, E is the Young’s modulus, d is the wire diameter, y is the cable deflection arrow, and L is the length of wire sample [29].

The radius of the guiding curve of the unpowered holder was chosen such that a 400 mm long specimen placed on the device would be shaped as close as possible to a circular slice. This configuration produced uniform tension throughout the length of the specimen. This device was equipped with a cycle counter offering a range of up to 99,999,999 cycles, a timer, and an electrical circuit that stopped the device when the sample (wire) broke. The red dots on the specimen correspond to the placement of the strain gauges to measure the actual stress present in the specimen.

**Figure 3:**
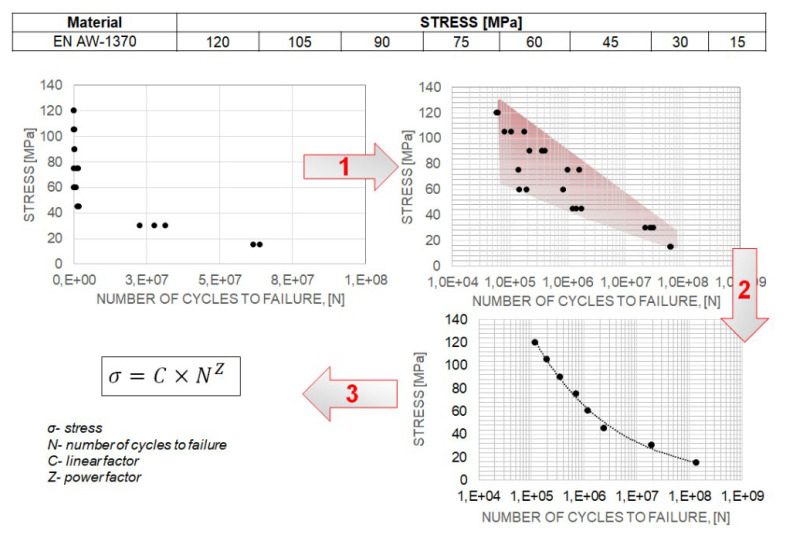
The methodology for analysing fatigue process results and constructing Wöhler curves.

The number of cycles to failure was measured three times for each stress level, and the average number of cycles was calculated. This average value was used to develop a Wöhler curve. Thus, determining a single Wöhler curve required about 30 measurements. Figure 3 presents the methodology for analysing fatigue process results and constructing Wöhler curves based on the fatigue process results.

The wires were tested to determine their structural properties. Microstructure tests were carried out using a scanning electron microscope (Hitachi model, Tokyo, Japan) with an accelerating voltage of 15 kV and an electron beam current of 30 nA.

## 3. Results and Discussion

The main objective of the present study was to determine the effects of mechanically hardening aluminium wires on the fatigue strength of such wires. The literature indicates that an increase in static strength leads to an increase in fatigue strength. The parameters and results of the fatigue test are shown in Table 3, including the mechanical properties and hardening states of the wires (in %), the maximum bending/rotating stress values during the fatigue tests, and the number of failure cycles.

**Figure 4:**
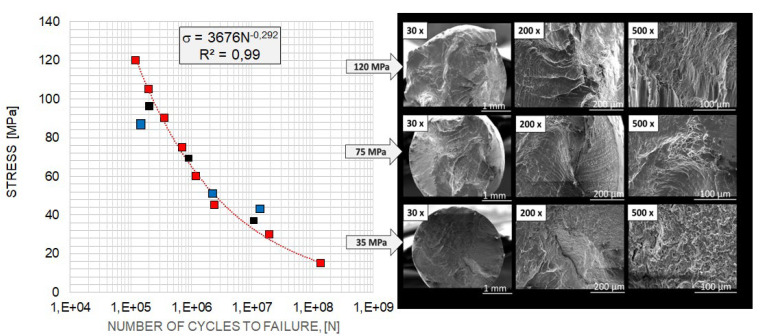
Wöhler curves of aluminium wire tested (red points on the left—test results; black, results in [23], blue—tested in [29]) along with SEM images of fatigue cracks of aluminium wire, magnification 100×.

The Wöhler characteristics provided the basis for the fatigue strength results. Figure 4 shows the Wöhler curves of aluminium wires under industrial conditions with a diameter of 3.0 mm (variant 1, engineering strain—90%). Our results were compared with those in the literature.

The black and blue dots represent, respectively, the test results for the aluminium wire studied in [7] and the wire studied in Nowak’s dissertation [30]. These results were similar to those of the present study. Different mechanisms of fatigue failure were observed depending on the stress level, as indicated by the nature of the fatigue cracks.

**Figure 5:**
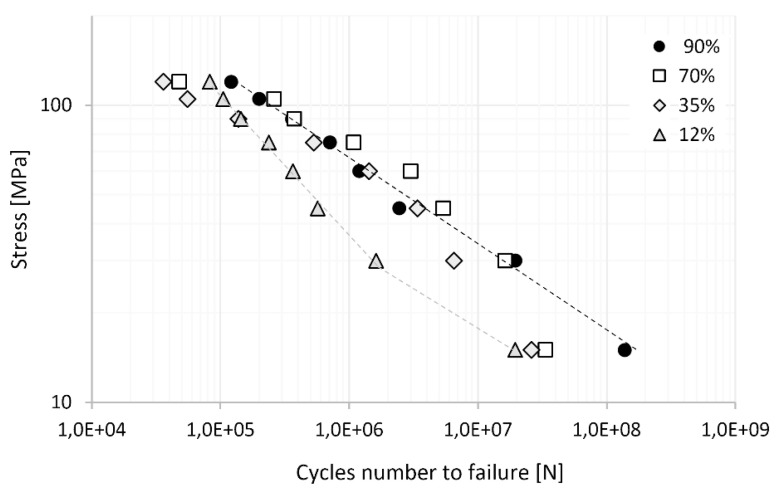
S–N curves of aluminium wires with different levels of strain hardening.

Figure 5 illustrates the Wöhler curves for aluminium wires with different levels of strain strengthening, i.e., 12, 30, 70, and 90%. We observed that an increase in reinforcement leads to an increase in fatigue strength by up to 1 order of magnitude. A detailed analysis of the results shows that the points form a straight line for the reinforced wires (black curve). However, an inflexion point was observed for the wires with the lowest reinforcement (12%). Additionally, the values of the C and Z parameters of the Wöhler curve were the highest among all analysed cases. The above result was due to the significant contribution of plastic deformation during the fatigue process, as a result of the high level of bending stress relative to the wire’s yield strength. Table 4 presents the results of aluminium wire’s fatigue strength, linear coefficient C, and power factor Z of Wöhler’s equation.

First, the wire in the state with the lowest quenching strain (12%) was clearly an outlier, where the C and Z coefficients showed significantly higher values compared to those in other cases. The high values of the C and Z coefficients indicate the low fatigue resistance of the wire in a near-annealed state, which is generally caused by the fatigue process occurring in a stress range higher than the yield stress, at which point permanent deformations occur. Under other levels of engineering strain (35, 70, and 90%), we found that the varying mechanical states of the wires translated into variation, especially in the value of linear coefficient C in the Wöhler equation, which varied by up to two times.

In the next stage of analysis, the fatigue crack morphology of the aluminium wires was analysed. In general, the nature of fatigue cracks depends on the type of material, the mechanical condition, and the level of material loading (stress) during the fatigue process.

**Figure 6:**
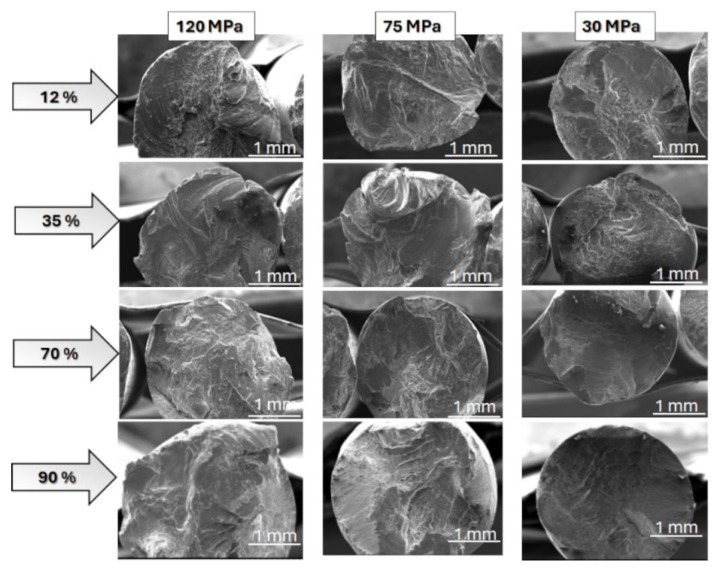
SEM images of the fatigue cracks of aluminium wires tested in this paper (rows show images of cracks in the wires with different levels of the hardening state, while columns indicate stress values during the fatigue test).

**Figure 7:**
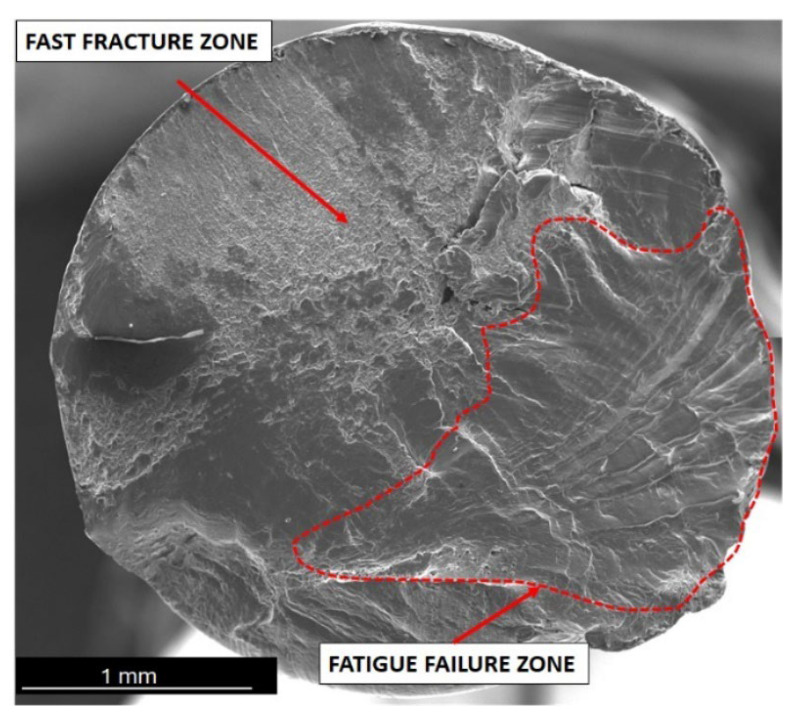
SEM image of aluminium (90% strain hardening) wire after fatigue tests (HCF), magnification 50×.

**Figure 8:**
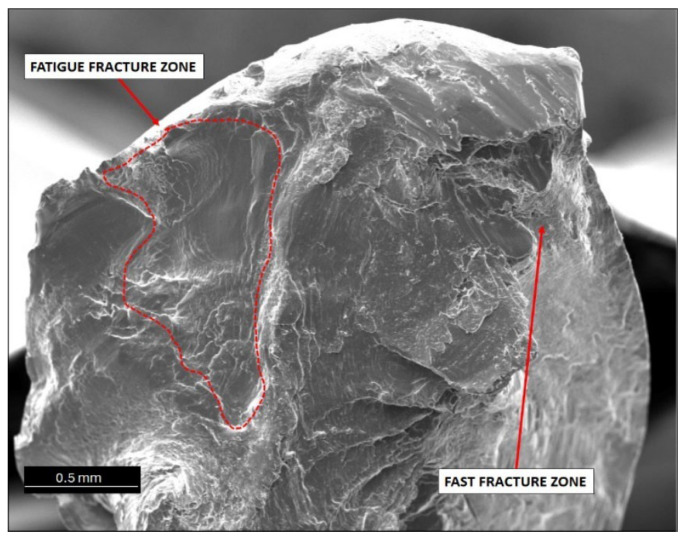
SEM image of aluminium wires after fatigue tests (LCF), magnification 50×.

**Figure 9:**
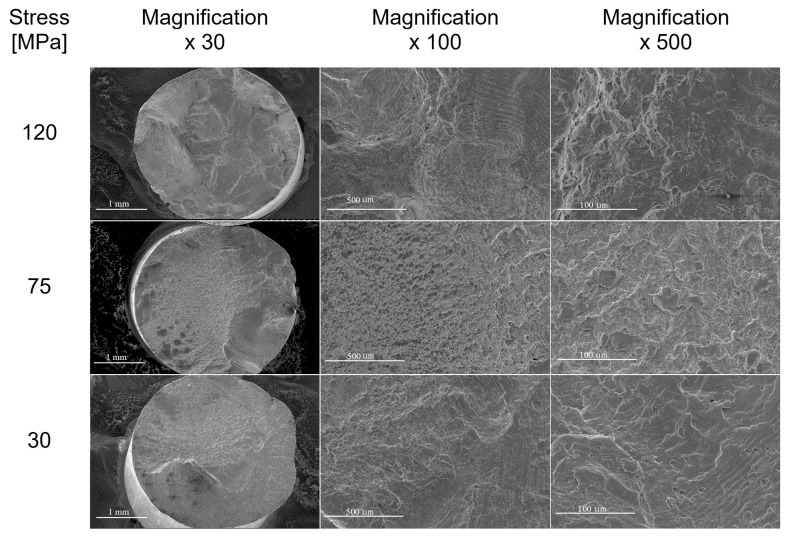
SEM images of fatigue failures of aluminium wires (engineering strain—90%). The rows show stress values during the fatigue test; columns—SEM images at different magnifications.

**Figure 10:**
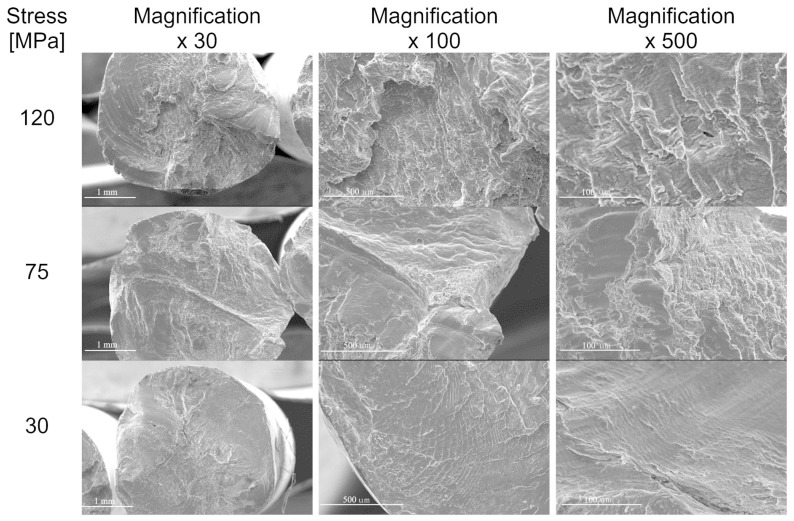
SEM images of the fatigue failures of aluminium wires (engineering strain—12%). The rows show stress values during the fatigue test; columns—SEM images at different magnifications.

The results of fatigue fracture of wires are presented in Figure 6, Figure 7, Figure 8, Figure 9 and Figure 10. Figure 6 shows a comparison of SEM images of fractures of all tested wires after fatigue testing at a magnification of 30×.

Generally, when analysing fatigue cracks, two characteristic areas can be distinguished: (a) the fatigue failure zone, where fatigue cracks begin and develop; and (b) the immediate failure zone, where material failure occurs. The fatigue failure zone is characterised by a smooth surface with visible lines and cyclic steps. However, the immediate failure zone is characterised by a developed surface with unevenly distributed cracks. This zone occurs just before the failure of the component.

One of the most critical areas in fatigue crack morphology is the so-called “focus”, where crack initiation occurs. In this area, the material experiences the highest stress concentration. Typically, the focus is surrounded by a prefocus zone, where macroscopic cracks can already be observed. Mutual cyclic friction between the surfaces of the prefocus zone during the fatigue process causes the surface to evolve, making it smooth and shiny. This evolution intensifies as the cyclic load on the material decreases during the fatigue process. In addition, the fatigue zone is characterised by the presence of steps known as fatigue lines.

The area of immediate failure is instead characterised by a much more developed surface, which is dynamically formed through a reduction in the cross-sectional area of the sample. This reduction is so significant that the specimen cannot carry further loads. Material destruction then occurs rapidly, often with a visible fracture zone [25,26,27,28].

Below are the results showing the influence of stress level in fatigue testing on the appearance of wires fractures using scanning electron microscopy. SEM image of fatigue fracture of a wire with engineering strain at a level of 90% subjected to fatigue under low stress (35 MPa) is shown in Figure 7. The same wire subjected to fatigue under high stress (120 MPa) is shown in Figure 10 (cf. Figure 4)

Fractographic examination revealed two distinct regions: a 40% smooth plane (upper region) and a 60% fibrous plane (lower region). Fatigue damage nucleated in an elliptical fretting trace. Details show a smooth fracture surface with prominent striations.

Fatigue failure initiates at the point of the highest stress concentration and then gradually develops, leading to a reduction in material cross section and, ultimately, a loss of load-bearing capacity and decohesion. Analysis of the SEM images of wires that failed during the initial phase of the fatigue cycle, corresponding to the highest bending stress, showed that in all cases, the cracks featured highly developed surfaces. Cracks were also dominated by areas with visible plastic deformation.

We also tested the impact of the hardened state of the wires on fatigue strength. The results showed that for wires with engineering strain to 90%, the fracture surface no longer showed dominant plastic deformation areas. In this case, typical fatigue crack morphology was observed, with parallel lines (striations) on the crack surface resulting from cyclic loading changes. The crack was dominated by a zone of crack initiation (the focus), a zone of fatigue failure, and a zone of immediate failure. Brittle fatigue areas were also observed. High stresses were designed to reveal surface defects, while low-cycle stresses (LCF) revealed structural defects.

Wires with engineering strain of 12% showed the most developed crack surfaces, which was attributed to the contribution of plastic deformation when the specimen was loaded (see Figure 10). Reduced bending stress changed the failure mechanism during the fatigue process. Analysis of the fatigue crack morphology of samples subjected to 120 MPa and 70 MPa loads revealed distinct and numerous degrees, extrusions, and bands of plastic fatigue.

## 4. Conclusions

Based on the experimental results, the following findings were obtained:

1. The variety of the material’s hardened states significantly affected its fatigue strength. While qualitatively obvious, these results required further in-depth quantitative evaluation. The results for the influence of strain hardening showed that among wires characterised by the lowest strain engineering (12%), the C and Z parameters had the highest values. This result was due to the significant contribution of plastic deformation during the fatigue process, given the high bending stresses relative to the yield strength of the wires.
2. Analysing the other test variants confirmed that an increase in wire hardenability led to an increase in resistance to cyclically applied stresses, resulting in smaller values for the linear and power coefficients in the Wöhler equation.
3. Analysis of the fatigue crack images demonstrated that different types of fatigue crack morphology can be observed depending on the mechanical conditions and stress levels. High stress levels resulted in cracks with highly developed surfaces dominated by plastic deformation, while low bending stress levels led to cracks with smooth surfaces covering nearly the entire wire cross section.

## Figures and Tables

**Table 1:** Chemical composition of aluminium (EN AW-1370) according to the standard and the aluminium wire rod used in the test.

Material	Chemical Composition [% by Weight].								
	Al min.	Si	Fe	Cu	Mn	Mg	Cr	Zn	Ga
EN AW-1370 in Accordance with the Standard	97.70	0.10	0.25	0.05	0.01	0.02	0.01	0.05	0.03
Aluminium Wire Used For Testing	97.78	0.03	0.10	0.04	0.002	0.001	0.001	0.03	0.01

**Table 2:** Mechanical properties of the aluminium wire rod and wires used in the tests.

Diameter d_0_	Diameter d_1_ [mm]	Engineering Strain	True Strain	UTS [MPa]	YP [MPa]	Elongation [%]
[mm]		[%]	[-]			
9.52	3.01	90	2.30	156	144	1.54
5.50	3.01	70	1.21	129	114	1.85
3.73	3.01	35	0.43	111	102	6.50
3.21	3.01	12	0.13	74	71	12.10

**Table 3:** Summary of mechanical properties of the tested aluminium wires and number of cycles to failure after the fatigue test under different stresses.

Variant	Engineering Strain [%]	UTS [MPa]	Yp [MPa]	Bending/Rotating Max. Stress [MPa]	Number of Cycles to Failure [N]
1	90	155	139	120	1.21 × 10^5^
				105	1.28 × 10^5^
				90	1.25 × 10^5^
				75	2.00 × 10^5^
				60	1.98 × 10^5^
				45	2.21 × 10^5^
				30	3.59 × 10^5^
				15	7.09 × 10^5^
2	70	129	116	120	1.20 × 10^6^
				105	6.44 × 10^6^
				90	2.36 × 10^7^
				75	1.98 × 10^8^
				60	4.80 × 10^4^
				45	2.61 × 10^5^
				30	3.75 × 10^5^
				15	1.08 × 10^6^
3	35	111	96	120	3.00 × 10^6^
				105	5.40 × 10^6^
				90	1.64 × 10^7^
				75	3.36 × 10^7^
				60	8.70 × 10^4^
				45	1.10 × 10^5^
				30	3.43 × 10^5^
				15	2.77 × 10^5^
4	12	74	69	120	8.07 × 10^5^
				105	5.10 × 10^6^
				90	3.09 × 10^6^
				75	2.60 × 10^7^
				60	8.25 × 10^4^
				45	1.05 × 10^5^
				30	1.44 × 10^5^
				15	2.37 × 10^5^

**Table 4:** Summary of linear coefficient C and power factor Z for wires with different levels of the hardening state.

Lp.	Die Angle 2α [°]	Engineering Strain [%]	C [-]	Z [-]	Wöhler Equation $\sigma =C\times N^{Z}$
1	16	90	3676	0.292	$\sigma =3676\cdot N^{-0.292}$
2	16	70	1871	0.239	$\sigma =1871\cdot N^{-0.239}$
3	16	35	2752	0.287	$\sigma =2752\cdot N^{-0.287}$
4	16	12	24,153	0.470	$\sigma =24153\cdot N^{-0.470}$

## Data Availability

The raw data supporting the conclusions of this article will be made available by the authors on request.
